# Penta­cyclo­[9.3.1.1^2,6^.1^4,8^.1^9,13^]octa­deca-1(2),8(9)-diene

**DOI:** 10.1107/S1600536812026785

**Published:** 2012-06-20

**Authors:** Savvas Ioannou, Eleni Moushi

**Affiliations:** a Chemistry Department, University of Cyprus, Nicosia 1678, Cyprus

## Abstract

The title compound, C_18_H_24_, was the main product of thermolysis of noradamantene dimer (hepta­cyclo­[9.3.1.1^2,6^.1^4,8^.1^9,13^.0^1,9^.0^2,8^]octa­deca­ne). The crystal structure was determined to prove that the thermolysis product of noradamantene dimer is favored by stretch release due to ring opening of the four-membered ring. The bond length of the quaternary C atoms of the starting material was calculated as 1.6 Å, enlarged in comparison to other single bonds. After the rearrangement, the stretch release of the above carbons leads to an increase of the distance between them (2.824 Å) with respect to the crystallographic data.

## Related literature

For reviews on noradamantene and analogous pyramidalized alkenes, see: Borden (1989 ▶, 1996 ▶); Vázquez & Camps (2005) ▶. For the syntheses of noradamantene dimer, see: Renzoni *et al.* (1986) ▶ and for related analogs, see: Camps *et al.* (1996*a* ▶,*b* ▶). For the synthesis of the precursor diiodide (3,7-diiodo-tricyclo-[3.3.1.0^3,7^]nona­ne), an important inter­mediate in the synthetic route towards the generation of noradamantene, see: Ioannou & Nicolaides (2009 ▶). For the synthesis of [2]diadamantane, see: McKervey (1980 ▶); Graham *et al.* (1973 ▶).
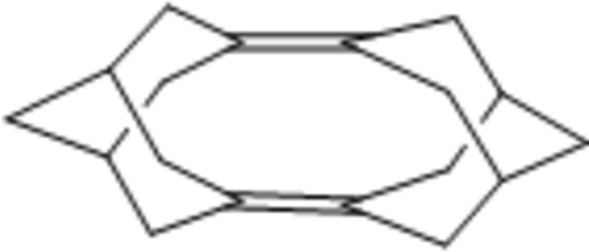


## Experimental

### 

#### Crystal data

C_18_H_24_
*M* *_r_* = 240.37
Orthorhombic, 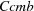

*a* = 8.5855 (6) Å
*b* = 15.6618 (10) Å
*c* = 9.3156 (6) Å
*V* = 1252.62 (14) Å^3^
*Z* = 4
Mo *K*α radiation
μ = 0.07 mm^−1^
*T* = 100 K
0.15 × 0.07 × 0.04 mm

#### Data collection

Oxford Diffraction SuperNova Dual (Cu) Atlas diffractometer
Absorption correction: multi-scan (*CrysAlis RED*; Oxford Diffraction, 2008 ▶) *T*_min_ = 0.530, *T*_max_ = 1.000
2341 measured reflections
640 independent reflections
514 reflections with *I* > 2σ(*I*)
*R*_int_ = 0.033

#### Refinement

*R*[*F* ^2^ > 2σ(*F* ^2^)] = 0.043
*wR*(*F* ^2^) = 0.117
*S* = 1.06
640 reflections
62 parameters
H atoms treated by a mixture of independent and constrained refinement
Δρ_max_ = 0.44 e Å^−3^
Δρ_min_ = −0.16 e Å^−3^

### 

Data collection: *CrysAlis CCD* (Oxford Diffraction, 2008 ▶); cell refinement: *CrysAlis CCD*; data reduction: *CrysAlis RED* (Oxford Diffraction, 2008 ▶); program(s) used to solve structure: *SHELXS97* (Sheldrick, 2008 ▶); program(s) used to refine structure: *SHELXL97* (Sheldrick, 2008 ▶); molecular graphics: *DIAMOND* (Brandenburg, 1999 ▶) and *Mercury* (Macrae *et al.*, 2006 ▶); software used to prepare material for publication: *WinGX* (Farrugia, 1999 ▶) and *publCIF* (Westrip (2010 ▶).

## Supplementary Material

Crystal structure: contains datablock(s) global. DOI: 10.1107/S1600536812026785/zj2078sup1.cif

Additional supplementary materials:  crystallographic information; 3D view; checkCIF report

## Figures and Tables

**Fig. 1.**
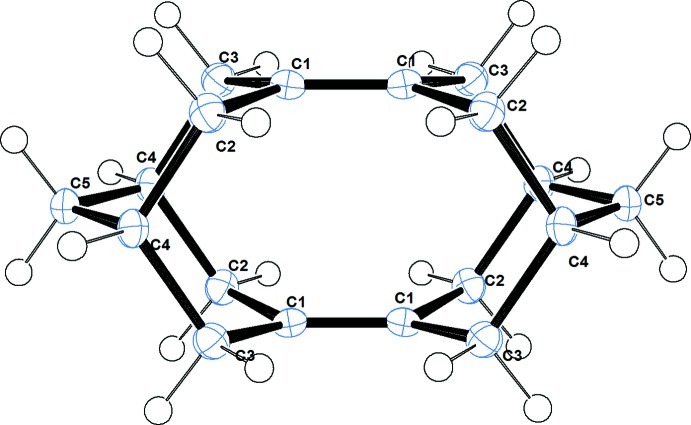
Structure of the title compound pentacyclo [9.3.1.12,6.14,8.19,13] octadeca-di-1(2),8(9)-ene with the atom-labelling. Displacement elipsoids are drawn at the 50% probability level.

**Fig. 2.**
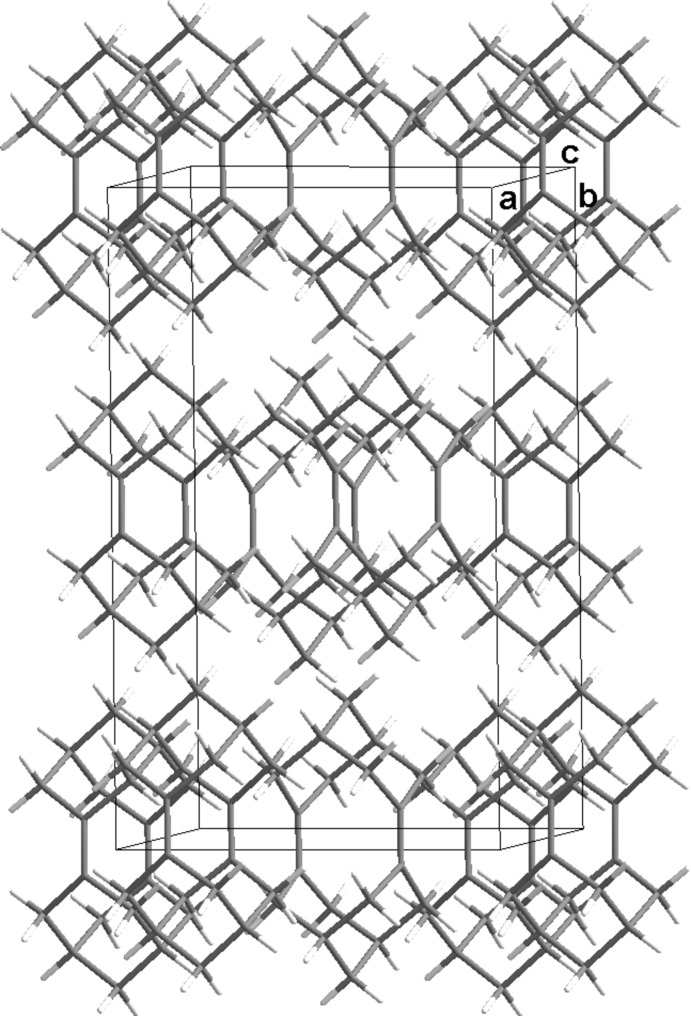
Molecular packing of the title compound, viewed along [1 0 0].

**Fig. 3.**
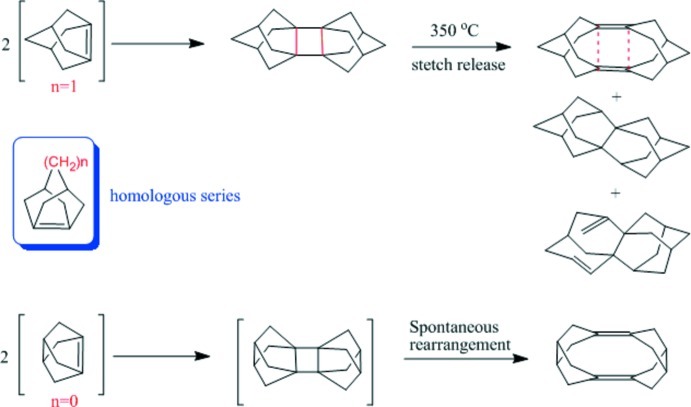
Dimer rearrangement of the two first members of a homologous series of pyramidalized alkenes.

**Fig. 4.**
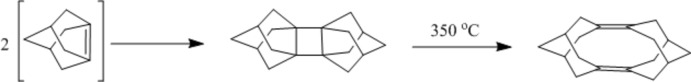
The formation of the title compound.

## supplementary crystallographic information

### Comment

Pyramidalized alkenes is a special category of olefins which have their four substituents of the double bond not lying on the same plane (Borden 1989, 1996, Vázquez & Camps, 2005). This fact makes the higher pyramidalized alkenes (like noradamantene) very reactive and impossible to isolate at ambient conditions. Due to their high reactivity, once they form, they react instantly with any nucleophile. In the absence of any reactive compound during their formation, the most common product is their [2 + 2] dimer. Noradamantene (n=1) is the second member of a homologous series of this category (figure 3) and it can serve as a building block for the formation of larger polycyclic hydrocarbons like the title compound. The most pyramidallized alkene (n=0) of the same homologous series is rearranged spontaneously to the corresponding diene once the dimer is formed (Camps *et al.* 1996a,b) (figure 3). This is attributed to its grater stretch due to the smaller carbon side chain. The title compound is the main product of thermolysis of noradamantene dimer and its formation depends on the reaction conditions. At different reaction conditions (higher temperatures, reaction time) [2]diadamantane (McKervey 1980, Graham *et al.* 1973) and another asymmetric diene were identified among the products.

### Experimental

Synthesis of pentacyclo [9.3.1.1^2,6^.1^4,8^.1^9,13^] octadeca-di-1(2),8(9)-ene. Heptacyclo [9.3.1.1^2,6^.1^4,8^.1^9,13^.0^1,9^.0^2,8^] octadecane(10 mg,0.042 mmol) was placed in a cylindrical glass container with small diameter (~5 mm suitable for glass workshops) sealed at the bottom edge, while the other edge was connected at the vacuum line. The glass cylinder was washed three times with argon and placed under vacuum for 5 minutes after which the opened edge was sealed as well with the use of a flamethrower, encapsulating the reactant under vacuum. The capsule was placed in a controlled temperature oven at 350 *^o^*C for 5 minutes. Crystals of the product and the reactant were formed when the capsule cooled down to room temperature. The starting material was removed by breaking carefully the glass of the one edge and washing the solid with hexane 3x1 ml. The residue was mostly product which was recrystallized by sealing the capsule again under vacuum and reheating it at 350°C for another 5 minutes. Colorless crystals of pure product were formed when the capsule cooled down to room temperature.

### Refinement

The H atoms are positioned with idealized geometry and refined using a riding model with *U*_iso_(H) = 1.2 of *U*_eq_ (C).

### Crystal data

**Table:**

C_18_H_24_	*F*(000) = 528
*M**_r_* = 240.37	*D*_x_ = 1.275 Mg m^−^^3^
Orthorhombic, *C**c**m**b*	Mo *K*α radiation, λ = 0.71073 Å
Hall symbol: -C 2bc 2bc	Cell parameters from 1022 reflections
*a* = 8.5855 (6) Å	θ = 3.4–28.9°
*b* = 15.6618 (10) Å	µ = 0.07 mm^−^^1^
*c* = 9.3156 (6) Å	*T* = 100 K
*V* = 1252.62 (14) Å^3^	Polyhedral, colorless
*Z* = 4	0.15 × 0.07 × 0.04 mm

### Data collection

**Table:**

Oxford Diffraction SuperNova Dual (Cu) Atlas diffractometer	640 independent reflections
Radiation source: SuperNova (Mo) X-ray Source	514 reflections with *I* > 2σ(*I*)
Mirror monochromator	*R*_int_ = 0.033
Detector resolution: 10.4223 pixels mm^-1^	θ_max_ = 26.0°, θ_min_ = 3.5°
ω scans	*h* = −10→9
Absorption correction: multi-scan (*CrysAlis RED*; Oxford Diffraction, 2008)	*k* = −19→18
*T*_min_ = 0.530, *T*_max_ = 1.000	*l* = −11→8
2341 measured reflections	

### Refinement

**Table:**

Refinement on *F*^2^	Primary atom site location: structure-invariant direct methods
Least-squares matrix: full	Secondary atom site location: difference Fourier map
*R*[*F*^2^ > 2σ(*F*^2^)] = 0.043	Hydrogen site location: inferred from neighbouring sites
*wR*(*F*^2^) = 0.117	H atoms treated by a mixture of independent and constrained refinement
*S* = 1.06	*w* = 1/[σ^2^(*F*_o_^2^) + (0.0549*P*)^2^ + 0.8624*P*] where *P* = (*F*_o_^2^ + 2*F*_c_^2^)/3
640 reflections	(Δ/σ)_max_ < 0.001
62 parameters	Δρ_max_ = 0.44 e Å^−^^3^
0 restraints	Δρ_min_ = −0.16 e Å^−^^3^

### Special details

**Table:**

Geometry. All e.s.d.'s (except the e.s.d. in the dihedral angle between two l.s. planes) are estimated using the full covariance matrix. The cell e.s.d.'s are taken into account individually in the estimation of e.s.d.'s in distances, angles and torsion angles; correlations between e.s.d.'s in cell parameters are only used when they are defined by crystal symmetry. An approximate (isotropic) treatment of cell e.s.d.'s is used for estimating e.s.d.'s involving l.s. planes.
Refinement. Refinement of *F*^2^ against ALL reflections. The weighted *R*-factor *wR* and goodness of fit *S* are based on *F*^2^, conventional *R*-factors *R* are based on *F*, with *F* set to zero for negative *F*^2^. The threshold expression of *F*^2^ > σ(*F*^2^) is used only for calculating *R*-factors(gt) *etc*. and is not relevant to the choice of reflections for refinement. *R*-factors based on *F*^2^ are statistically about twice as large as those based on *F*, and *R*- factors based on ALL data will be even larger.

### Fractional atomic coordinates and isotropic or equivalent isotropic displacement parameters (Å^2^)

**Table:**

	*x*	*y*	*z*	*U*_iso_*/*U*_eq_	Occ. (<1)
C1	0.11524 (14)	0.04287 (9)	0.10813 (13)	0.0158 (4)	
C2	0.00121 (16)	0.09715 (9)	0.19231 (15)	0.0180 (4)	
C3	0.20910 (17)	0.09726 (9)	0.00491 (15)	0.0178 (4)	
C4	−0.10310 (16)	0.15159 (9)	0.09316 (15)	0.0177 (4)	
C5	0.0000	0.20830 (13)	0.0000	0.0190 (5)	
H5A	0.0641	0.2446	0.0603	0.023*	0.50
H5B	−0.0641	0.2446	−0.0603	0.023*	0.50
H2A	−0.0646 (17)	0.0622 (11)	0.2559 (18)	0.024 (4)*	
H2B	0.0618 (18)	0.1376 (11)	0.256 (2)	0.032 (4)*	
H3A	0.2779 (19)	0.1360 (11)	0.0608 (17)	0.028 (4)*	
H3B	0.2842 (18)	0.0618 (11)	−0.0514 (15)	0.023 (4)*	
H4	−0.1732 (16)	0.1880 (9)	0.1554 (15)	0.012 (3)*	

### Atomic displacement parameters (Å^2^)

**Table:**

	*U*^11^	*U*^22^	*U*^33^	*U*^12^	*U*^13^	*U*^23^
C1	0.0144 (7)	0.0184 (7)	0.0147 (7)	0.0009 (5)	−0.0028 (5)	0.0008 (6)
C2	0.0205 (8)	0.0174 (8)	0.0161 (7)	0.0012 (6)	0.0007 (6)	−0.0008 (6)
C3	0.0162 (7)	0.0180 (8)	0.0192 (8)	−0.0013 (6)	0.0001 (6)	0.0012 (6)
C4	0.0192 (7)	0.0140 (7)	0.0201 (7)	0.0025 (5)	−0.0002 (6)	−0.0043 (6)
C5	0.0223 (10)	0.0120 (10)	0.0228 (10)	0.000	−0.0051 (8)	0.000

### Geometric parameters (Å, º)

**Table:**

C1—C1^i^	1.343 (3)	C3—H3B	1.000 (16)
C1—C2	1.5153 (18)	C4—C5	1.5250 (17)
C1—C3	1.5164 (18)	C4—C3^ii^	1.5450 (19)
C2—C4	1.5434 (18)	C4—H4	1.012 (14)
C2—H2A	0.984 (17)	C5—C4^ii^	1.5250 (17)
C2—H2B	1.009 (18)	C5—H5A	0.9700
C3—C4^ii^	1.5450 (19)	C5—H5B	0.9700
C3—H3A	0.994 (17)		
			
C1^i^—C1—C2	124.13 (7)	H3A—C3—H3B	103.3 (13)
C1^i^—C1—C3	124.17 (7)	C5—C4—C2	109.00 (11)
C2—C1—C3	110.88 (11)	C5—C4—C3^ii^	109.04 (11)
C1—C2—C4	112.03 (11)	C2—C4—C3^ii^	113.04 (11)
C1—C2—H2A	111.7 (10)	C5—C4—H4	110.1 (8)
C4—C2—H2A	109.5 (9)	C2—C4—H4	108.3 (8)
C1—C2—H2B	108.7 (9)	C3^ii^—C4—H4	107.4 (8)
C4—C2—H2B	107.6 (10)	C4—C5—C4^ii^	108.75 (15)
H2A—C2—H2B	107.0 (15)	C4—C5—H5A	109.9
C1—C3—C4^ii^	111.80 (11)	C4^ii^—C5—H5A	109.9
C1—C3—H3A	109.1 (9)	C4—C5—H5B	109.9
C4^ii^—C3—H3A	108.9 (9)	C4^ii^—C5—H5B	109.9
C1—C3—H3B	111.3 (9)	H5A—C5—H5B	108.3
C4^ii^—C3—H3B	112.1 (9)		

Symmetry codes: (i) *x*, −*y*, *z*; (ii) −*x*, *y*, −*z*.
